# Operational Efficiency and Liquidity Within Hurricane Prone Hospitals in the United States: A Regional Study

**DOI:** 10.5430/ijfr.v17n1p27

**Published:** 2026-01-23

**Authors:** George Audi, Hanadi Hamadi, Margaret Capen, Rima Tawk

**Affiliations:** 1 Associate Professor, School of Allied Health Sciences-Division of Healthcare Management, Florida A&M University, USA; 2 Professor, Department of Health Administration, Brooks College of Health, University of North Florida, USA; 3 Professor, College of Business, East Carolina University, USA; 4 Professor, College of Pharmacy and Pharmaceutical Sciences/Institute of Public Health, Florida A&M University, USA

**Keywords:** natural disasters, financial liquidity, hospital closure

## Abstract

Natural disasters, in particular hurricanes, are linked to hospitals’ financial performance, how various parameters affect hospitals’ financial metrics, and financial indicators that are extremely relevant in establishing hospitals’ sustainability in any eventual emergency (Mah & Andrew, 2022; Schick et al., 2025). Most notably, hurricanes have led to the shutting down of hospitals in rural settings because rural health systems, especially hospitals, have unique issues when they face disasters (Traynor, 2020; Desai et al., 2019). Days Cash on Hand is a key financial indicator used to mark a hospital’s capability to recover from a financial crunch, such as a calamity. Data were obtained from the 2023 American Hospital Association (AHA) Annual Survey. The Bonferroni multiple comparisons test reveals that government-owned hospitals keep 101.75 days’ worth of cash on hand, whereas private hospitals keep 40.75 days’ worth of cash on hand. This results in government hospitals holding 61 more days of cash on hand than their private counterparts.

## 1. Introduction

The frequency of natural disasters, particularly hurricanes, is increasing. Therefore, studying hospitals’ financial performance and healthcare delivery is essential, especially in vulnerable regions throughout the United States (Espinel et al., 2019). Financial ratios such as days cash on hand (DCOH) and operating margins are benchmarks that hospital financial officers and management use to gather essential information to ensure a hospital’s financial stability, especially during a disaster (Pai & Park, 2025; Reiter et al., 2015). The escalating frequency and intensity of natural disasters, mostly hurricanes, have become a significant threat to the United States. Such disasters destroy an area’s infrastructure and environment and adversely affect all aspects of the healthcare system in the affected area. Hospitals are at the bedrock of care for the populations they serve and must confront a twofold painful challenge: meeting the imminent needs of populations affected by natural disasters with quality healthcare and securing their own financial well-being.

Natural disasters, in particular hurricanes, are linked to hospitals’ financial performance, how various parameters affect hospitals’ financial metrics, and financial indicators that are extremely relevant in establishing hospitals’ sustainability in any eventual emergency (Mah & Andrew, 2022; Schick et al., 2025). Most notably, hurricanes have led to the shutting down of hospitals in rural settings because rural health systems, especially hospitals, have unique issues when they face disasters (Traynor, 2020; Desai et al., 2019). The pertinence of disaster planning in maintaining continuity of care provision and hospitals’ fiscal stability is a key element of hospital solvency, especially in above-area-risk locations. Over the past few decades, natural disasters have been particularly frequent and intense in the Southern United States, a fact that may be explained by phenomena such as weather patterns and environmental degradation to a significant degree. The occurrence of such disasters as wildfires, floods, hurricanes, and major storms has become more frequent, and most importantly, the economic dismantling that they pose must be considered. Natural disasters have become the cause of increasing economic losses in the U.S., amounting to billions of dollars on an annual basis (Botzen, 2019; Lou & Buntin, 2025).

As the number of natural disasters and weather patterns continue to increase, scientists have predicted an even more spectacular display of weather-related incidents, which will cost more and lead to more population distress. Specifically, most low-income and rural areas, in terms of population and increasingly aging infrastructure, are the most marginalized demographics in the US population with respect to natural disasters. These regions are most likely to suffer disproportionately compared to their counterparts due to their poor capacities for preparing for and recovering after natural disasters (Iglesias et al., 2021).

## 2. Literature Review

### 2.1 Hurricane Risks in the US

Hurricanes, which are ranked as one of the worst forms of natural disasters in the U.S., have been reported to have increased in frequency and intensity in the past few years. Based on the research findings, Atlantic hurricanes have been growing stronger, with many major hurricanes occurring in the past few decades (Hertelendy et al., 2024; (Vecchi et al., 2021). For example, in 2023, the Atlantic hurricane season became the most active in history, and several storms caused significant damage (Bercos-Hickey, 2024). These trends highlight the need to enhance disaster preparedness and resilience, particularly in regions prone to hurricanes. Because more severe storms are more likely to occur, healthcare and related services in these areas must adapt to these rising natural events.

A wide range of factors affects hospital profitability in the external and internal fields, such as the number of patients, reimbursement rates, personnel size, and operational efficiency (Tonna et al., 2020). In hospitals, revenue generation is largely correlated with the number of patients and how they are treated. Many patients, especially in emergencies amidst disasters, may produce higher revenue levels, but at the same time, this means that the hospital’s resources will be overwhelmed. Moreover, hospitals that can deliver high-quality care and do not incur costs are more likely to achieve better financial results (Beauvais et al., 2023; Lalani et al., 2023). A hospital’s patient–payer mix is associated with private and government schemes, such as Medicare and Medicaid, and is an important factor in the hospital’s profitability. These reimbursements, specific to geographical area and payer-mix type, determine how much hospitals can receive from a single patient. Hospitals in regions where the percentage of uninsured or underinsured people is higher can experience greater financial stress, especially in the middle of and after the disaster, when demand for healthcare services grows (Beauvais et al., 2023).

### 2.2 Effects of Hurricanes on Hospitals

Hurricanes are among the most destructive natural disasters, and they affect hospitals’ operations in many ways. After a hurricane, hospitals must suddenly act as emergency centers and shelters while dealing with damage to their buildings and operations (Melnychuk et al., 2022). Strong winds and floods can destroy buildings and disrupt power, water, and transportation, negatively affecting the logistics associated with supplies, staff, and patients. Power outages are especially detrimental to hospital operations because hospitals constantly require electricity to support life, use machines for diagnosis, and run operations. Many hospitals have backup generators, but these often do not supply enough power and can run out of fuel or become damaged by floods.

In addition to causing physical damage, hurricanes place excessive strain on hospital staff and resources (Masbi et al., 2024). Following a hurricane, emergency rooms become crowded with patients with injuries from the storm, long-term illnesses worsen, and people who have become unhoused seek refuge in hospitals. People who need treatments such as dialysis or chemotherapy come to hospitals when their regular clinics are closed. At the same time, hospital employees might be dealing with personal difficulties, such as damaged homes or being forced to leave the area. This results in fewer staff (nurses, doctors, support staff, etc.) when the hospital needs them the most. Hospitals might also have to arrange large evacuations if the buildings are unsafe, which involve significant planning, moving patients to other facilities, and finding enough transportation, often when things are chaotic (Haghpanah et al., 2021).

Hurricanes can also affect hospital finances. Damage to buildings, loss of money from canceled surgeries, and caring for uninsured disaster victims can all reduce profits. Even after the storm passes, hospitals may face issues with repairs, supply shortages, and staffing for weeks or months. In places that are often hit by hurricanes, including the United States Gulf Coast and the Southeast, these repeated events can seriously harm long-term finances. Some of these effects occur long after the storm, but the impact of hurricanes on hospitals is significant, affecting safety, staffing, and financial stability.

### 2.3 Uniqueness of Hospitals in the U.S. Economy

Hospitals differ in the U.S. economy because they are a mix of public service, private business, and important community hubs (Jeurissen et al., 2021). Unlike many businesses, hospitals offer important services all day, every day that cannot stop during a crisis. They are key to our country’s health system, mainly providing acute care, special treatments, and emergency help. The need for hospitals is constant, regardless of whether the economy is bad or an environmental disaster occurs. This sets them apart from other areas of the economy that stop or decrease productivity. One factor that makes hospitals unique is how they are paid and the rules they follow. In the U.S., they receive money from a mix of sources: private insurance, Medicare, Medicaid, and patients paying themselves (Eschliman et al., 2023). Payments are often set by the government and contracts, not the usual market prices. This implies that hospitals cannot easily change prices when costs increase or decrease. They must also follow many federal and state rules pertaining to patient safety, reporting quality, staffing, and preparing for emergencies. The mix of set prices and rules creates a unique financial and operational situation

Hospitals are also large employers and help local economies. They are often the largest or one of the largest single employers in a region. Besides direct employment, hospitals and their employees buy from local businesses and help the area grow. Many hospitals are nonprofit organizations that are meant to benefit the community by providing free or reduced-cost care or investing in public health (Zare et al., 2021). Private hospital systems usually serve as vital anchors in smaller or rural areas. Hospitals also have high fixed costs; buildings, gear, and specialized staff are all fixed costs. This makes them vulnerable to sudden disruptions to their business models, but it also indicates that they are designed for the public’s greater good. Owing to their social role, unique funding, and economic importance, hospitals often cannot be subjected to the same business models as other areas of the economy. Hospital workers are caregivers, employers, teachers (with residency programs), and first responders (Choyke et al., 2023). This special role makes talking about their financial future even more important because if a hospital closes, it negatively affects the health and local economy of the population that it serves.

### 2.4 Importance of Operating Margins for Hospitals

The operating margin shows how well a company turns sales into profit before paying interest (Evans, 2004). If the margin is high, the business can pay for its operations (such as staff, materials, rent, and marketing) and still have money left over to reinvest, give to owners, or get through tough times. Businesses in competitive markets can use a strong operating margin to change prices, invest in new ideas, or grow without needing to borrow significantly. The main goal of most businesses is to maximize owner wealth. Owners and investors focus on margins to see how well the management is performing and how well the company is doing compared to its competitors. A shrinking margin could mean that costs are increasing, prices are not aligned with costs, or operations are not running smoothly. On the other hand, a consistently high margin can attract investors, raise stock prices, and help the company secure better deals with suppliers and lenders. Therefore, the operating margin is a financial number and a way to measure a company’s standing against its competitors.

The operating margin is crucial when addressing potential issues. If a company’s margins are small, it is more at risk from problems such as supply chain problems, rising costs, recessions, and other unforeseen events. Companies may not have sufficient funds to survive economic downturns, possibly leading to layoffs, budget cuts, or even shutdowns. However, companies with healthy profit margins usually weather the storm, retain their workers, and may even take business from struggling competitors. This ability to recover is required in any business model because industries can change quickly or require significant investment because changes in cash flow can threaten a company’s long-term survival. Operating margins also affect a company’s ability to innovate and grow. Companies that are doing well can invest in research and innovation and expand into new markets or business lines. Financial institutions and investors look at these margins to see how risky (beta) a company is when performing due diligence before extending capital. Decent margins make a company look less risky, which could mean lower interest rates and easier access to funds for the company. Therefore, it is imperative to examine how financially sound an organization is, how well it is being managed, and how ready it is to address unforeseen challenges.

Although the theoretical basis of operating margin is the same for hospitals as it is for other companies, there are some differences between the two. The operating margin is key for all types of industries even though it appears a bit different for the healthcare industry. A hospital’s operating margin, which is the difference between what it earns and what it spends, is a way to see how well it is doing financially (Gaffney & Michelson, 2023). It is important for hospitals to keep providing effective care, improve their facilities, and handle any urgent situations. Hospitals must address rising costs in terms of salaries, medicine, and updated equipment (Hinrichs-Krapels et al., 2022). At the same time, what insurance companies pay them does not always increase at the same rate, which affects their profits. If a hospital has a healthy operating margin, it can pay for operational improvements, including new services, better diagnostic tools, and electronic health records (Ly et al., 2011). These adjustments help patients by increasing safety, improving quality, and making the organization run more smoothly. If hospitals do not make enough money, they might defer repairs, cut staff, and/or offer fewer services, ultimately hurting patients and the patient population.

Having sufficient capital reserves allows hospitals to store emergency supplies, maintain backup power systems, and train staff for disasters. Operating margins also affect a hospital’s borrowing capacity. If a hospital desires to obtain a loan, lenders assess its ability to pay it back. Poor margins can mean higher loan rates or difficulty in obtaining loans, which makes investing in better care harder. In rural and poor areas, where hospitals already operate on tight budgets, a financial crisis, such as a storm, pandemic, or change in insurance payments, can force a hospital to close (Keesee et al., 2024). Moreover, operating margins are tied to a hospital’s purpose. Nonprofit hospitals need to give back to the community through free or reduced-cost services, which are subsidized by the profits that they obtain through their normal business lines. If margins shrink, hospitals might decrease community investments when people need them the most. Therefore, the operating margin is not just an arbitrary financial figure; it is imperative to understand a hospital’s overall health along with its ability to serve during turbulent financial times. Ultimately, a healthy operating margin indicates to investors that a hospital can bounce back, discover new ideas, and take care of its community.

### 2.5 DCOH and Its Importance to Hospitals

Days Cash on Hand (DCOH) is a key financial indicator used to mark a hospital’s capability to sail through a financial crunch, such as a calamity. This indicator is described as the number of days a hospital can use only its liquid assets to cover its operating expenses without receiving extra income. For example, hospitals in hurricane-prone regions enjoy stronger DCOH because of the necessary cushion to cover unforeseen costs, such as fixing and repairing infrastructure, supplementing their workforce, and purchasing medical supplies (Tsuboi et al., 2021; Zinoviev et al., 2021). A larger DCOH implies that a hospital is more capable of dealing with its revenues; in other words, it can continue providing its services in emergencies.

DCOH is a key financial indicator used to mark the capability of a hospital to sail through a financial crunch, like a calamity. This indicator is described as the number of days a hospital can use only its liquid assets to cover its operating expenses without getting an extra income. A larger DCOH implies that such a hospital is more capable of dealing with its revenues; in other words, it can continue providing its services in emergencies (Beauvais et al., 2023). One of the major advantages of DCOH is that it provides a financial cushion during emergencies. Hospitals face unexpected circumstances, such as natural disasters, pandemics, and influxes of patients. Increased DCOH acts as a backup when the regular revenue stream is disrupted because hospitals will have enough funds to cover operating expenses, including repair costs, salaries, and other medical supplies (Blavin et al., 2023). During the COVID-19 pandemic, for example, hospitals with better DCOH were able to absorb the need to expand the security of ICUs and overtime pay without necessarily fearing the immediate loss of revenue.

Another benefit of DCOH is that it increases hospitals’ financial stability. Hospitals with better DCOH are more able to absorb shocks in their finances and continue important services without having to reduce them. These hospitals do not need to resort to external borrowing or make radical decisions, including staff or service cuts, to survive (Handlon et al., 2025). Low-DCOH hospitals, on the other hand, are more susceptible to financial turmoil in periods of low revenues, which may result in the disruption of services or financial failure. DCOH assists hospital leadership in proper planning and is a vital part of future decisions that the organization will make.

A healthy DCOH can provide financial freedom to hospitals by allowing them to concentrate on growth and quality of care delivery and invest in new technologies without concern about the current financial implications. With the ability to predict short-term losses, hospital administrators can make better-informed decisions concerning budget allocation, debt management, and resource utilization, knowing that they have a safety net to help them survive temporary problems. Moreover, financial institutions and investors, for a more stable hospital, can promote better financing options for future expansion and development (Ganzach, 2000). Thus, hospitals will have the capacity to sustain themselves during economic downturns and therefore will be able to grow, innovate, and be competitive in the healthcare industry.

## 3. Data and Methodology

### 3.1 Data Collection

Data were obtained from the 2023 American Hospital Association (AHA) Annual Survey. The 2023 AHA Annual Survey Dataset captured the hospital name, address, zip code, Days Cash on Hand, and operating margin. The states that were selected for the analysis included: Alabama, Florida, Georgia, Kentucky, Louisiana, Mississippi, North Carolina and South Carolina as these have been that states that have seen the highest amount of hurricane activity in recent history. The analysis excluded Veteran Affairs (VA) hospitals, as they do not report financial information to the CMS.

### 3.2 Analysis Techniques

An initial examination for possible correlation between the financial variable, days cash on hand, and operating margin, virtually no multivariate correlation as measured by variance inflation factors of approximately 1. Therefore, although the use of multiple dependent variables would usually suggest the use of a MANCOVA model, the lack of a sufficient degree of correlation (r values considered to be between .3 and .9, or comparable VIFs between 1.43 and 10) necessitates the use of three separate analyses of covariance (ANCOVA) models instead. These models were run using a GLM modeling procedure because of unmatched cells in the treatment variable (ownership).

An analysis of covariance procedure evaluates whether population means of a dependent variable are the same for all levels of a categorical independent variable while controlling for the effects of a quantitatively measured covariate(s), NAPCT. The means (LS Means) of the dependent variable were adjusted to what they would be if the treatment groups were equal for all covariates. The treatment effect variable in the model is hospital ownership status. (i.e., whether the hospital is government or non-government owned) a for-The covariate, hurricane risk (NAPCT) is a percentile measure of the hurricane loss risk of a particular hospital’s zip code relative to the national risk loss and varies between 0 and 1. There were 1032 hospitals in the dataset, of which 147 were critical access hospitals.

The null hypotheses are as follows:

H1: No significant difference exists in the DCOH between government and non-government-owned hospitals.

H2: There is no significant difference in the operating margin between government and non-government-owned hospitals.

## 4. Results

The results are presented in two tables on the following page. Overall, both models were significant, with p values of <.0001. The tables on the following page show the individual F-tests for the treatment and covariate variables in each model. Table 1 shows the SAS results of the ANCOVA model for cash-on-hand. The treatment variable, hospital ownership type (OWN), was significant at a p-value (pr>F) of <.0001. The covariate NAPCT was insignificant (pr>F=.1989). Least square means are then computed for the government-owned hospitals (1) and private (0) groups. The Bonferroni multiple comparisons test reveals that government-owned hospitals keep 101.75 days’ worth of cash on hand, whereas private hospitals keep 40.75 days’ worth of cash on hand. This results in government hospitals holding 61 more days of cash on hand than their private counterparts.

Table 2 shows the results of the ANCOVA model with the operating margin as the dependent variable. It also shows that the government/private hospital status effect is significant (pr>F<.0001). The covariate NAPCT was not significant at p = .5892. The LS means show an average operating margin of −51.34% for government-owned hospitals and 1.18% for private hospitals, a difference of 52.52%. Over half of the government-owned hospitals in the sample had negative operating margins.

The assumptions underlying the models were also tested. The homogeneity of variances was confirmed, as was the independence of the effect (government/private) and covariate variables. The former was verified by plotting the residuals of the model against the predicted values. To check for independence of the treatment and covariate variables, an interaction term between the two was included in the models. As the interaction term was insignificant in both models, it was removed, and proof of independence was established. The only assumption violated was the multivariate normality assumption. Tests of normality revealed p values of <.01 for both models. All outliers were removed from the original data; therefore, the non-normality in models 1 and 2 was caused by slight skewness in the data. Because the number of observations in each treatment group exceeded 30 and non-normality was not severe (as also seen in the plot of the data), the results should be somewhat robust.

## 5. Discussion

The healthcare industry is always changing and adapting to provide the medical care that its population requires. This has led to healthcare managers finding themselves having to adapt their business practices to stay in business and serve their communities (Foo et al., 2024; Rivera-Diaz et al., 2024). People do not simply stop utilizing medical services when a natural disaster strikes an area or they lose their ability to pay. The opposite occurs when a community is disrupted, and the need for and utilization of emergency medical services increases (Chakravorty & Knapp, 2019). This highlights the importance of hospitals monitoring their financial and operational benchmarks and increasing their awareness of threats originating from national disasters (Stewart, 2019). With increased hurricane activity, especially in southern states, hospitals need to ensure that they can stay solvent during times when their businesses could be partially or fully disrupted (Hertelendy et al., 2024) (Sands et al., 2022).

Ensuring that a hospital has adequate financial reserves to meet its daily capital expenditures is imperative to ensure that the organization stays solvent, especially during challenging times, such as a hurricane or other natural disasters (Masbi et al., 2024; Olschewski, 2024). A hospital’s operating margin is widely viewed as a broad indication of the overall health of an organization’s business model. In many cases, government-owned hospitals have unique funding structures and financial safety nets that they can access that their private counterparts might not have access to. This is especially important during challenging times, such as during hurricanes and other national disasters (Karim et al., 2025). Non-operating revenue (NOR), derived from government appropriations, can contribute to financial stability of hospitals by offsetting operating losses and improving profitability when traditional funding models would not be sufficient due to a hospital being taken offline due to a natural disaster (Pitcher et al., 2024). Hospitals, especially, rural hospitals are disproportionately facing financial liquidity challenges based on their declining profitability resulting in their increased risk of for closure. Hospitals that have stable funding streams, alternative funding streams, and/or or governmental funding have more access to funding sources which could be beneficial in times of natural disasters when a hospital is in part or totally taken offline (Bai & Anderson, 2016). Hospitals are seen as critical to the overall security and strength of the communities they serve (Foo et al., 2024). This is especially true for vulnerable segments of the community. If a hospital closes, the entire population it serves is negatively impacted in perpetuity.

## 6. Conclusion

The economic reality for many hospitals in the Southern United States is that they are in a hurricane-prone area known as “Hurricane Alley,” along with having to constantly monitor and adapt to liquidity and operational issues. This could cause a problem for many hospitals, not just in hurricane-prone areas but in any region in the United States that experiences a higher-than-normal risk of natural disasters, such as flooding, earthquakes, tornadoes, and even extreme weather. In our initial discovery on this topic, the researchers noted how little of the United States was not susceptible to one or more of these natural disasters.

In many cases, a region may be susceptible to multiple natural disasters, such as hurricanes and flooding. It is also important to note, as we saw during the 2024 flooding in Western North Carolina that resulted from the remnants of Hurricane Helene, that although the area did not experience hurricane-force winds, the unprecedented rainfall led to catastrophic flooding for the affected population.

Researchers should examine how hospitals in areas that are more prone to natural disasters account for disruptions to their business, along with whether there has been an organizational funding structure that has increased the hospital’s visibility and financial viability after a major natural disaster.

## Figures and Tables

**Table 1.** ANCOVA for Dependent Variable: Days Cash-on-Hand (n=894)

Source	Df	Type III SS	Mean Square	F value	PR>F
OWN	1	597752.5282	597752.5282	17.38	<.0001
NAPCT	1	56841.1590	56841.1590	1.65	.1989
Least Squares Means for Cash-on-Hand					
Ownership	LS Mean				
1 (Government)	101.754658				
0 (Private)	40.746216				

**Table 2.** ANCOVA for Dependent Variable: Operating Margin (n=1011)

Source	Df	Type III SS	Mean Square	F value	PR>F
OWN	1	496906.9288	496906.9288	30.90	<.0001
NAPCT	1	4668.0305	4668.0305	.29	.5892
Least Squares Means for Operating Margin					
Ownership	LS Mean				
1 (Government)	−51.3436750				
0 (Private)	1.1833762				

## Data Availability

The data that support the findings of this study are available on request from the corresponding author. The data are not publicly available due to privacy or ethical restrictions.

## Data sharing statement

No additional data are available.

## Abbreviations

The following abbreviations are used in this manuscript:

DCOH: Days Cash on Hand
ICU: Intensive Care Unit
AHA: American Hospital Association
VA: Veterans Administration
CMS: Center for Medicare and Medicaid Services
NAPCT: National Hurricane Percentile Risk per NOAA
NOAA: National Oceanic and Atmospheric Administration
